# Molecular insights into ROS-induced aggregation in chicken primordial germ cells and its alleviation by ascorbic acid

**DOI:** 10.1186/s40104-026-01521-9

**Published:** 2026-11-09

**Authors:** Kexin Wu, Zheyi Jiang, Qiulian Wang, Bowen Niu, Xiqiong Wang, Shenghan Gao, Dandan Sun, Ruoyu Yang, Congjiao Sun, Ning Yang

**Affiliations:** 1 State Key Laboratory of Animal Biotech Breeding, Frontier Science Center of Molecular Design Breeding, China Agricultural University, Beijing, 100193 China; 2 National Engineering Laboratory for Animal Breeding, College of Animal Science and Technology, China Agricultural University, Beijing, 100193 China

**Keywords:** Antioxidants, Ascorbic acid, Cellular senescence, Chicken primordial germ cells, Oxidative stress

## Abstract

**Background:**

Primordial germ cells (PGCs) are highly valuable for chicken genetic engineering; however, their cultivation is frequently hindered by spontaneous cell aggregation and slow proliferation in certain breeds.

**Methods:**

We systematically evaluated the culture dynamics of PGCs derived from four distinct chicken breeds. To investigate the molecular mechanisms driving these phenotypes, we performed integrated transcriptomic and proteomic profiling, alongside targeted exogenous supplementation assays.

**Results:**

We found that White Leghorn PGCs exhibited superior line establishment efficiency, whereas broiler breeds, particularly White Cornish, displayed severe cell aggregation and delayed growth. Transcriptomic and proteomic analyses revealed that WC PGCs exhibit an inherent deficiency in glutathione metabolism and an activation of the p53 signaling pathway. This finding was experimentally corroborated by the accumulation of reactive oxygen species (ROS) in these cells. Based on these mechanistic insights, we experimentally demonstrated that targeted exogenous supplementation with L-sulforaphane and ascorbic acid effectively mitigated excessive ROS and ameliorated the aggregation phenotype in White Cornish PGCs, with ascorbic acid notably accelerating cell proliferation. Subsequently, ascorbic acid also promoted proliferation in both Rhode Island Red and White Plymouth Rock lines, while alleviating aggregation in the White Plymouth Rock line.

**Conclusions:**

These findings demonstrate the cross-breed applicability of ascorbic acid, suggesting it could serve as a general supplement for PGC culture in other chicken breeds. Collectively, our study proposes a mechanistic model in which oxidative stress promotes PGC aggregation and offers practical antioxidant interventions to enhance the derivation efficiency of chicken PGCs, providing a valuable reference for the future optimization of in vitro culture media.

**Graphical Abstract:**

**Figure:**
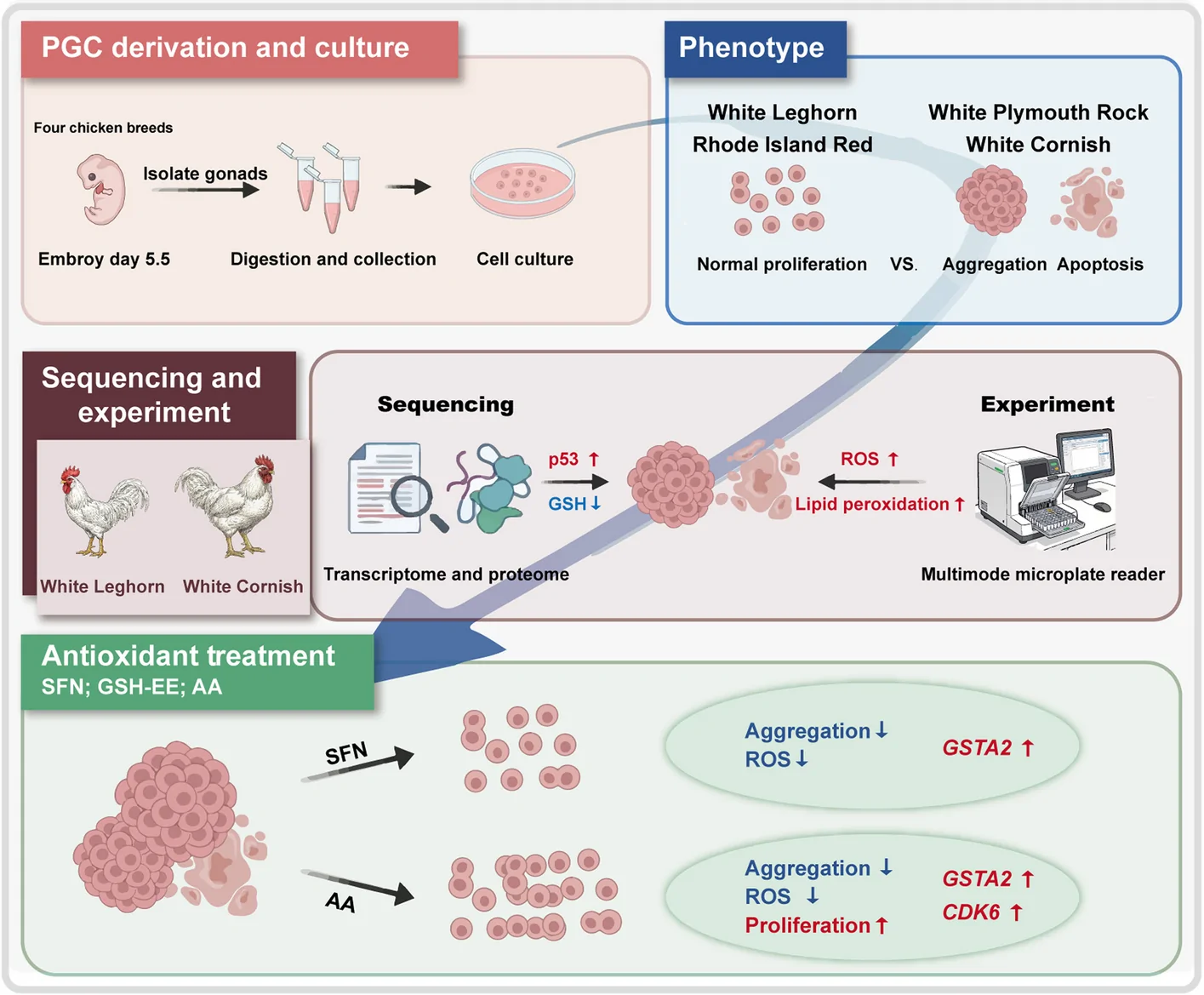

**Supplementary Information:**

The online version contains supplementary material available at https://doi.org/10.1186/s40104-026-01521-9.

## Introduction

The chicken represents a uniquely versatile and invaluable organism in modern biological sciences. They are not only excellent model organisms for fundamental research on developmental biology, immunology, and infectious diseases, but also economically vital agricultural animals that are essential for meeting the increasing demand for high-quality dietary protein [1–5]. Driven by the rapid expansion and intensification of the commercial poultry industry, the demand for effective disease management, disease-resistant breeding, and comprehensive germplasm conservation is becoming increasingly pressing [6–9]. Meeting these multifaceted challenges necessitates innovative genetic strategies that transcend the limitations of conventional practices. While historically successful, traditional selective breeding programs are incredibly time-consuming and are rapidly approaching physiological and genetic bottlenecks. Consequently, chicken primordial germ cell (PGC)-mediated gene editing and germplasm cryopreservation have emerged as pivotal next-generation biotechnologies with transformative potential [10]. These advanced approaches provide novel and accelerated pathways for precise avian genetic improvement, the generation of bioreactor flocks, and the critical conservation of rare or endangered genetic lineages [11–13].

As obligate precursors to adult gametes, PGCs are foundational to embryonic development and reproductive continuity [14]. In contrast to mammalian PGCs, which undergo interstitial migration through the hindgut and dorsal mesentery, avian PGCs migrate via the embryonic blood circulation to colonize the gonadal ridge [15, 16]. Specifically, avian PGCs originate from the epiblast and area pellucida, enter the vasculature during early somitogenesis, and subsequently migrate into the developing gonads [17, 18]. Leveraging this highly accessible pathway, researchers can readily isolate PGCs from either embryonic blood or gonadal tissues for in vitro expansion and long-term culture [19]. This provides the possibility for gene editing, as genomic modifications (such as CRISPR/Cas9 editing) require stringent clonal selection and extensive cellular proliferation, which typically necessitates one to two months of continuous in vitro maintenance. These genetically modified PGCs are subsequently injected into the vasculature of sterile surrogate embryos. Upon reaching sexual maturity and mating, these surrogate hosts efficiently produce donor-derived offspring, enabling the successful generation of genetically engineered chicken flocks [20–26].

While the aforementioned pipeline has proven highly successful in practice, efficient de novo derivation and long-term in vitro culture of stable PGC lines remain challenging. Under optimal conditions, chicken PGCs exist as phase-bright, suspension cells (typically 10–20 μm in diameter) that exhibit robust pluripotency and self-renewal. However, removing these cells from their tightly regulated embryonic niche and placing them in artificial in vitro culture microenvironments frequently induces severe cellular stress. This stress manifests as abnormal, large-scale physical aggregation, sudden proliferation impairment, and subsequent widespread apoptosis [27]. These detrimental phenotypes severely impede the establishment and translational application of PGC lines, particularly those derived from commercially valuable genetic backgrounds. Although in vitro culture-induced oxidative stress is hypothesized to trigger this aggregation and growth stagnation [28], the precise downstream molecular mechanisms remain poorly understood. To address this issue, decades of intense artificial selection have drastically altered the basal metabolic rates of different chicken breeds, namely, layers and broilers, potentially influencing their in vitro stress responses. While these breed-specific discrepancies have been extensively studied [29], their manifestation in PGCs remains largely unexplored. Specifically, there is a lack of systematic multiomics studies detailing how these differences dictate divergent cell fates during in vitro derivation [30, 31]. Consequently, we hypothesize that continuous selective breeding has profoundly reshaped the intrinsic metabolic profiles of PGCs.

Here, following the morphological observation that broiler-derived PGCs are highly susceptible to severe physical aggregation, we performed integrated transcriptomics and proteomics to systematically evaluate the molecular differences among in vitro cultured PGCs derived from two layer breeds (White Leghorn, WL; Rhode Island Red, RIR) and two broiler breeds (White Cornish, WC; White Plymouth Rock, WPR). Specifically, comparative analysis using WC and WL as representative models revealed that the aggregation phenotype is closely associated with oxidative stress induced by the dysregulation of glutathione (GSH) metabolism and p53 signaling. To gain these mechanistic insights, we evaluated targeted antioxidant interventions. Our results demonstrate that alleviating ROS accumulation, particularly through ascorbic acid (AA) supplementation, not only resolves cell aggregation but also enhances proliferation in multiple breeds of PGCs. Ultimately, this study elucidates the critical role of oxidative stress in PGC culture dynamics and offers a broadly applicable antioxidant strategy to optimize the in vitro culture of chicken PGCs.

## Materials and methods

### Experimental animals

All animal experiments were conducted according to the ethical policies and procedures approved by the Institutional Animal Care and Use Committee of China Agricultural University (Issue No. AW31506202-1-03). To establish the in vitro culture system for PGCs, fertilized eggs from four distinct chicken breeds including White Leghorn, Rhode Island Red, White Cornish, and White Plymouth Rock were selected for embryonic incubation. The fertilized eggs of the WL breed were obtained from the Poultry Genetic Conservation and Breeding Experimental Station of China Agricultural University (Beijing, China). The eggs of the remaining three breeds (RIR, WC, and WPR) were sourced from Beijing Yukou Poultry Industry Co., Ltd. (Beijing, China).

### PGC derivation and culture

Fertilized eggs were incubated for 5.5 d, after which the embryonic gonads were dissected. During dissection, the gonads from each embryo were collected individually and then processed as a single biological sample per embryo, which was cultured independently. Following enzymatic dissociation, PGCs were isolated and cultured as previously described [32]. Briefly, the main components of the cell culture medium were as follows: Avian KO-DMEM basal medium, 100 µmol/L β-mercaptoethanol (Gibco, Waltham, MA, USA, Cat. No. 21985023), 100 µmol/L Sodium Heparin (Sigma-Aldrich, USA, Cat. No. H3149), 10 µg/mL Ovotransferrin (Sigma-Aldrich, USA, Cat. No. C7786), 0.2% Ovalbumin (Sigma-Aldrich, USA, Cat. No. A5503), 4 ng/mL rhFGF (R&D Systems, USA), 30 ng/mL Activin A (PeproTech, Rocky Hill, NJ, USA), 1 × B-27 supplement (Gibco, Waltham, MA, USA, Cat. No. 17504). Cells were cultured in PGC medium, which was refreshed daily. A PGC line was defined as stably proliferating once the total cell number reached 1 × 10^5^ and the line could be continuously maintained in vitro for several months. To eliminate potential confounding effects of sex‑specific differences on proliferation and aggregation, all biological replicates (three per group) in this study were derived from male PGC lines.

### DNA extraction and sex identification

Genomic DNA was isolated from the collected PGCs utilizing a DNA Extraction Kit (TIANGEN, Beijing, China, Cat. No. YDP304). PCR-based sex determination was conducted by targeting the avian *CHD1* gene with specific primers (Table S4). The resulting PCR amplicons were subjected to agarose gel electrophoresis to identify the sex of the cell lines based on their characteristic banding profiles (a single band for ZZ males and double bands for ZW females).

### Cell proliferation assay

Proliferation dynamics were assessed using the CCK-8 assay (Beyotime, Shanghai, China, Cat. No. C0038). Exponentially growing cells were seeded in 96-well plates at 4,000 cells/well (three biological replicates; three technical replicates per group). Every 24 h, cells were incubated with 10 μL of CCK-8 reagent in 100 μL of medium for 1 h at 37 °C in the dark. Absorbance was recorded at 450 nm and 650 nm (reference). After excluding data outliers to obtain a mean from three representative replicates, the relative proliferation rate was determined. Specifically, the reference-corrected OD values (A_450_ − A_650_) of the experimental samples were background-subtracted using blank wells, and the resulting net absorbance at each 24 h interval was normalized against the initial net absorbance at 0 or 24 h. The normalized data were subsequently analyzed using Dunnett's test.

### RNA sequencing

Total RNA was isolated using the TRIzol Reagent (Thermo Fisher Scientific, Waltham, MA, USA, Cat. No. 15596018), after which the concentration, quality and integrity were determined using a NanoDrop spectrophotometer (Thermo Fisher Scientific, Waltham, MA, USA). Three micrograms of RNA were used as input material for the RNA sample preparations. Sequencing libraries were generated according to the following steps. Firstly, mRNA was purified from total RNA using poly-T oligo-attached magnetic beads. Fragmentation was carried out using divalent cations under elevated temperature in an Illumina proprietary fragmentation buffer. First strand cDNA was synthesized using random oligonucleotides and Super Script II. Second strand cDNA synthesis was subsequently performed using DNA Polymerase I and RNase H. Remaining overhangs were converted into blunt ends via exonuclease/polymerase activities and the enzymes were removed. After adenylation of the 3′ ends of the DNA fragments, Illumina PE adapter oligonucleotides were ligated to prepare for hybridization. To select cDNA fragments of the preferred 400–500 bp in length, the library fragments were purified using the AMPure XP system (Beckman Coulter, Beverly, CA, USA). DNA fragments with ligated adaptor molecules on both ends were selectively enriched using Illumina PCR Primer Cocktail in a 15 cycle PCR reaction. Products were purified (AMPure XP system) and quantified using the Agilent high sensitivity DNA assay on a Bioanalyzer 2100 system (Agilent). The sequencing library was then sequenced on a NovaSeq X Plus platform (Illumina) at Shanghai Personal Biotechnology Co. Ltd.

### RNA-Seq data processing and expression quantification

Raw sequencing data in FASTQ format were initially subjected to quality control using fastp (v0.22.0) [33] to remove adapter sequences and low-quality reads, thereby generating high-quality clean data. The resulting clean reads were subsequently aligned to the reference genome *Gallus gallus* (GRCg6a) using HISAT2 (v2.1.0) [34]. For gene expression quantification, HTSeq (v0.9.1) was employed to calculate the raw read counts mapped to each gene. Finally, the expression levels were normalized utilizing the FPKM (Fragments Per Kilobase of transcript per Million mapped reads) metric.

### Differential expression gene analysis

Difference expression of genes was analyzed by DESeq2 (v1.50.2) [35] with screened conditions as follows: expression difference multiple |log_2_FC| ≥ 1 or |log_2_FC| ≥ 0.58, significant *P*-value < 0.05.

### Differential expression protein analysis

Following peptide quantification, samples were analyzed using a Vanquish Neo UHPLC system coupled to an Orbitrap Astral mass spectrometer (Thermo Fisher Scientific, Waltham, MA, USA) at Majorbio Bio-Pharm Technology Co., Ltd. (Shanghai, China). Mass spectrometry data were acquired in data-independent acquisition (DIA) mode. Protein and peptide identification criteria were set to a false discovery rate (FDR) ≤ 0.01, with a peptide confidence level ≥ 99% and an extracted ion chromatogram (XIC) width ≤ 75 ppm. For quantitative analysis, missing intensity values were imputed using the sequential k-nearest neighbors (seqKNN) algorithm. Following log_2_ transformation, Student's *t*-tests were performed, and DEPs were defined based on thresholds of *P* < 0.05 and |log_2_FC| ≥ 0.58 or |log_2_FC| ≥ 0.26.

### Enrichment analysis

We mapped all the genes to Terms in the Gene Ontology database and calculated the numbers of differentially enriched genes in each Term. Using topGO (v2.50.0) to perform GO enrichment analysis on the differential genes (up DEGs/down DEGs), calculate *P*-value by hypergeometric distribution method (the standard of significant enrichment is *P*-value < 0.05), and find the GO term with significantly enriched differential genes to determine the main biological functions performed by differential genes. ClusterProfiler (v4.18.4) [36] software was used to carry out the enrichment analysis of the KEGG pathway of differential genes, focusing on the significant enrichment pathway with *P*-value < 0.05. The GO and KEGG pathway enrichment analyses for the proteomic data were performed using the same methods as described above for the transcriptomic data.

### Intracellular ROS quantification

ROS levels were evaluated using a ROS Assay Kit (Beyotime, Shanghai, China, Cat. No. S0033). Collected PGCs were incubated with a 1:1,000 dilution of DCFH-DA probe at 37 °C for 30 min in the dark. Fluorescence intensity was subsequently recorded using a microplate reader (Ex/Em = 488/525 nm). From the initial five replicates per group, anomalous outliers were removed, and the average of the remaining three values was normalized to the baseline control. Data analysis and graph generation were executed utilizing R (version 4.5.2) or GraphPad Prism (version 8.4.3) software.

### Measurement of lipid peroxidation levels

Lipid peroxide levels were measured using the BODIPY 581/591 C11 probe from a commercial kit (Beyotime, Shanghai, China, Cat. No. S0043). The collected cells were incubated with a 1:1,000 dilution of the 2 mmol/L probe stock at 37 °C for 30 min in the dark. After washing out the excess probe, dual-wavelength fluorescence was recorded using a microplate reader. The relative magnitude of lipid peroxidation was determined by calculating the Red/Green fluorescence intensity ratio.

### Formulation of antioxidants

L-Sulforaphane (Sigma-Aldrich, USA, Cat. No. S6317) was reconstituted in DMSO as a 50 mmol/L stock and diluted in culture medium to working concentrations of 0.2, 0.5, 1, and 5 μmol/L. GSH-EE (Sigma-Aldrich, G1404) and ascorbic acid (Sigma-Aldrich, A8960) were dissolved in sterile nuclease-free water to generate 50 mmol/L and 150 mmol/L stock solutions, respectively. For dose–response assays, both aqueous stock solutions were diluted in culture medium to final concentrations ranging from 0.2 to 10 mmol/L (0.2, 0.5, 1, 2, 5, and 10 mmol/L).

### Dose–response screening

To assess proliferative capacity and oxidative status, three independent biological replicates of WC PGCs were centrifuged, resuspended in PBS, and subjected to a second centrifugation. The resulting cell pellets were then resuspended in either the standard PGC culture medium or the PGC medium supplemented with antioxidants to their respective working concentrations. Subsequently, the cells were seeded into 48-well plates (30,000 cells/well, in triplicate) and cultured for 48 h without medium replacement. Post-incubation, intracellular ROS accumulation was measured via a DCFH-DA probe, while total cell counts and viability were determined using an automated cell counter equipped with the AO/PI method. Statistical comparisons were conducted using Dunnett's test. Data analysis and visualization were executed using R software (version 4.5.2) with the ggplot2 package [37].

### Morphological observation of PGCs

For comparative morphological evaluation, three independent male PGC lines (biological replicates) derived from WL and WC breeds were plated into 24-well plates. Cells were seeded at 60,000 cells per well in triplicate (technical replicates) and cultured in their respective complete media. At 48 h post-seeding, the phenotypic states of the in vitro cultured PGCs were systematically examined, and representative bright-field images were acquired using an inverted microscope equipped with a 10× objective.

### RNA extraction and RT-qPCR analysis

Total RNA was extracted from PGCs using TRIzol reagent (Invitrogen, Waltham, MA, USA, Cat. No. 15596018CN) following a standard chloroform-isopropanol precipitation protocol. The purified RNA was resuspended in sterile nuclease-free water, quantified via a NanoDrop spectrophotometer (Thermo Fisher Scientific, Waltham, MA, USA), and stored at −80 °C. First-strand cDNA was synthesized using the PrimeScript™ RT reagent Kit with gDNA Eraser (Takara Bio Inc., Kusatsu, Japan, RR047). Subsequent quantitative real-time PCR (qPCR) was performed to assess target gene expression, utilizing *GAPDH* as the internal control for normalization (Table S4). The assays were executed in triplicate for both biological and technical replicates. Relative gene expression was determined using the 2^−ΔΔCt^ method, with expression levels normalized against the WL-CT group. A one-way analysis of variance (ANOVA) followed by Tukey’s post hoc test was utilized.

### Statistical analysis

All experiments were performed with three biological replicates and three technical replicates. All results are expressed as mean ± standard deviation (SD). Statistical evaluation of the qPCR data was conducted utilizing GraphPad Prism software (version 8.4.3) and R software (version 4.5.2). For comparisons between two independent groups, an unpaired Student's *t*-test was applied. For comparisons involving three or more groups, a one-way analysis of variance (ANOVA) followed by Tukey’s post hoc test was utilized. Significance thresholds were established as ^*^P < 0.05, ^**^P < 0.01, and ^***^P < 0.001.

## Results

### Breed-specific landscapes of proliferation and physical aggregation in chicken PGCs

**Fig. 1:**
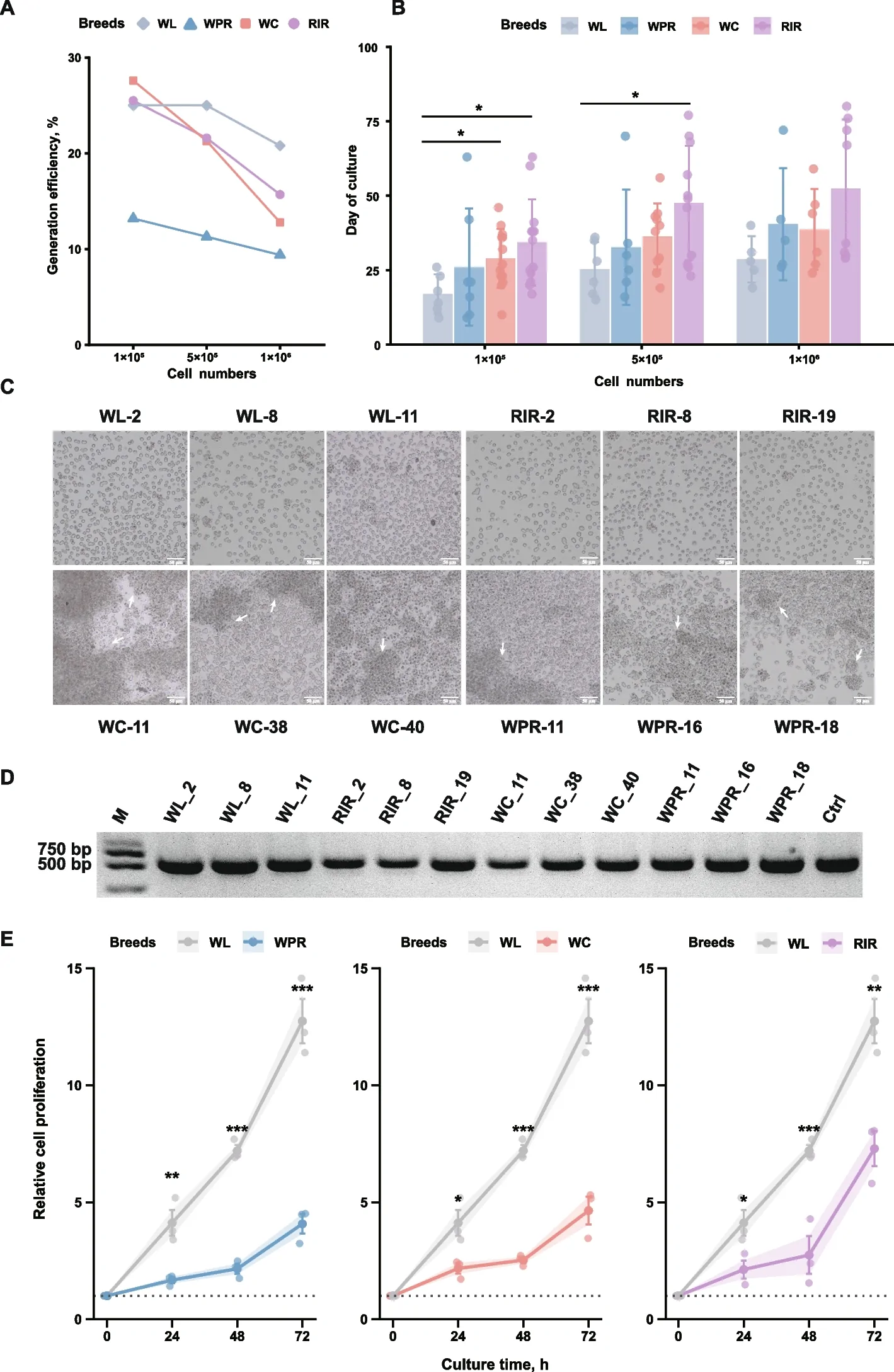
Derivation efficiency, morphological characteristics, and proliferation dynamics of PGCs from four chicken breeds. **A** In vitro establishment efficiency of PGC lines derived from WL, RIR, WC, and WPR breeds. **B** Time required (in days) for the diverse PGC lines to reach a defined population threshold during continuous culture. **C** Representative morphological images of the four PGC lines following long-term in vitro cultivation (~120 d). Scale bar = 50 μm. **D** PCR-based sex identification of three established PGC lines for each breed utilized for subsequent multi-omics sequencing (Ctrl: Male cell). **E** Cell viability and proliferation curves of the four PGC lines at 0, 24, 48, and 72 h, as determined by the CCK-8 assay. Data are presented as the mean ± SD (*n* = 3 independent biological replicates). ^*^P < 0.05, ^**^P < 0.01, and ^***^P < 0.001 compared with the WL group

Given the potential differences regarding in vitro establishment efficiency and proliferation rates across distinct breeds, we sought to conduct a more comprehensive evaluation. Hence, PGCs were obtained and cultured from the gonads of ED5.5 embryos from two layer breeds (WL, RIR) and two broiler breeds (WC, WPR). Based on microscopic observations and routine growth monitoring, we determined that WL PGCs achieved the highest efficiency of line establishment. In contrast, although WC PGCs initially exhibited rapid proliferation and a relatively high success rate, many of these lines suffered from severe cell aggregation and subsequent apoptosis during prolonged in vitro culture. This impaired normal proliferation and prevented further expansion, ultimately causing a drastic decline in the final efficiency of line establishment (Fig. 1A and C). Subsequent statistical analysis (*t*-test) of the growth data further demonstrated that WL PGCs required the shortest duration to proliferate to the target population size, whereas RIR PGCs took the longest (Fig. 1B).

To avoid confounding from sex‑specific differences and ensure more stable growth dynamics during culture, we selected three male PGC lines with comparable in vitro establishment times (30–40 d) from each breed (Fig. 1D) for CCK-8 assays and all subsequent comparative analyses across breeds. Given the superior efficiency and rapid growth of WL PGCs established above, this breed was utilized as the baseline control for comparison. The CCK-8 results were highly consistent with our assessments of line establishment and cell yield. WL PGCs exhibited the most stable and robust proliferation rate, while WC and WPR PGCs were the slowest among these four breeds. Notably, RIR PGCs only displayed a significant proliferative trend after 48 h of culture. This delayed onset indicates a prolonged doubling time, perfectly corroborating the phenotypic observation that RIR PGCs require the longest duration to reach the target cell mass (Fig. 1E).

Collectively, these findings confirm that WL PGCs achieve the highest efficiency of line establishment and the most rapid population doubling, prompting us to use this breed as the standard control in all subsequent analyses.

### Transcriptome profiling reveals a divergence between WC and WL PGCs

**Fig. 2:**
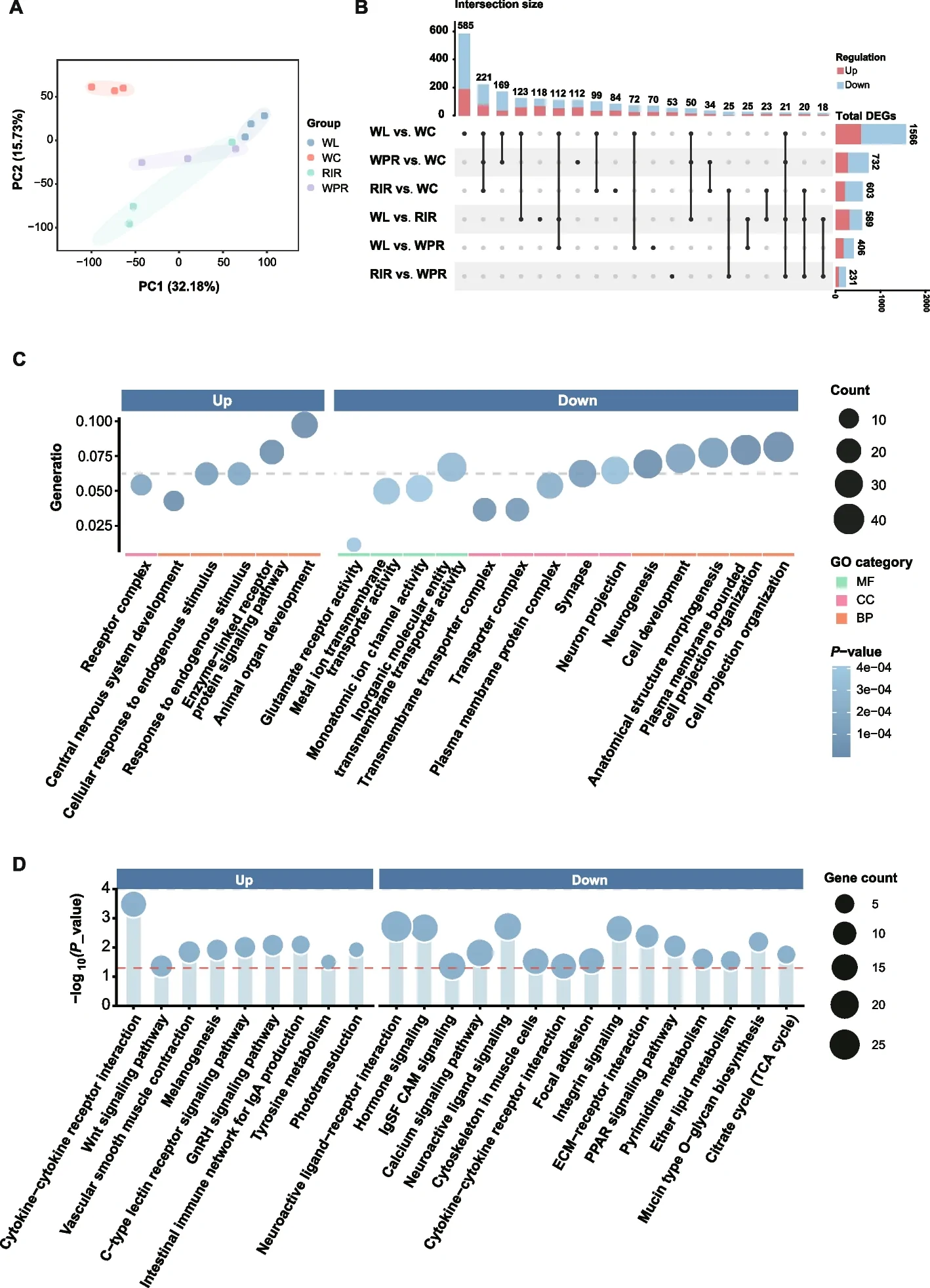
Transcriptomic profiling reveals differential antioxidant capacities between WL and WC PGCs. **A** PCA score plot of the global transcriptomic profiles from WL and WC groups. **B** UpSet plot illustrating the distribution of DEGs filtered by the thresholds of |log_2_FC| ≥ 1 and *P* < 0.05. **C** GO enrichment analysis of DEGs between WC and WL. The top 5 significantly enriched terms (*P* < 0.05) categorized by Biological Process (BP), Cellular Component (CC), and Molecular Function (MF), ranked by gene count, are displayed. **D** KEGG pathway enrichment analysis. The top 15 significantly enriched pathways (*P* < 0.05) for both up-regulated and down-regulated DEGs are presented separately based on gene counts

To explore the molecular basis underlying the observed disparities in the efficiency of line establishment, proliferation, and aggregation phenotypes among the distinct PGC lines, we performed RNA-seq on 12 established male lines (30–40 d to derivation) after approximately 120 days of in vitro culture. Principal component analysis (PCA) demonstrated four clusters, with WL and WC displaying the most pronounced spatial divergence (Fig. 2A). Additionally, we conducted differential gene expression analysis among the four breeds. Differentially expressed genes (DEGs) were subjected to UpSet plot analysis (filtered by *P* < 0.05 and |Log_2_FC| ≥ 1) to extract the top 20 intersection subsets. The WC vs. WL comparison generated the most extensive differential expression profile, yielding 575 upregulated and 991 downregulated genes, alongside 585 uniquely regulated DEGs (Fig. 2B and Fig. S1A). Based on these results, WL and WC were chosen for in-depth mechanistic exploration.

Separate GO enrichment analyses of the upregulated and downregulated DEGs revealed profound transcriptional remodeling in WC PGCs relative to WL PGCs (Fig. 2C). Specifically, receptor signaling cascades were robustly activated; upregulated DEGs were significantly enriched in “receptor complex”, “enzyme-linked receptor protein signaling pathway”, and “response to endogenous stimulus”, suggesting that WC PGCs actively attempt to sense environmental cues to cope with culture-induced stress. Concurrently, functions related to membrane transport and cell projection were broadly repressed. Downregulated DEGs clustered densely in pathways critical for membrane extensibility and molecular exchange, such as “transmembrane transporter complex”, “ion channel activity”, and “cell projection organization”, indicating highly abnormal membrane dynamics in WC PGCs.

Subsequent separate KEGG enrichment analyses of the upregulated and downregulated DEGs revealed a profound suppression of energy metabolism in WC PGCs compared to WL PGCs (Fig. 2D). Specifically, downregulated DEGs were significantly enriched in core energy and macromolecular metabolic hubs, including the “TCA cycle”, “PPAR signaling pathway”, and “lipid metabolism”. Overall, the extensive transcriptomic divergence establishes WC and WL as the two phenotypic extremes. The concurrent shutdown of core energy networks and activation of receptor signaling cascades suggest that WC PGCs reside in a state of severe cellular stress and metabolic crisis during in vitro culture.

### Proteomic signatures uncover metabolic and redox imbalance in WC PGC lines

**Fig. 3:**
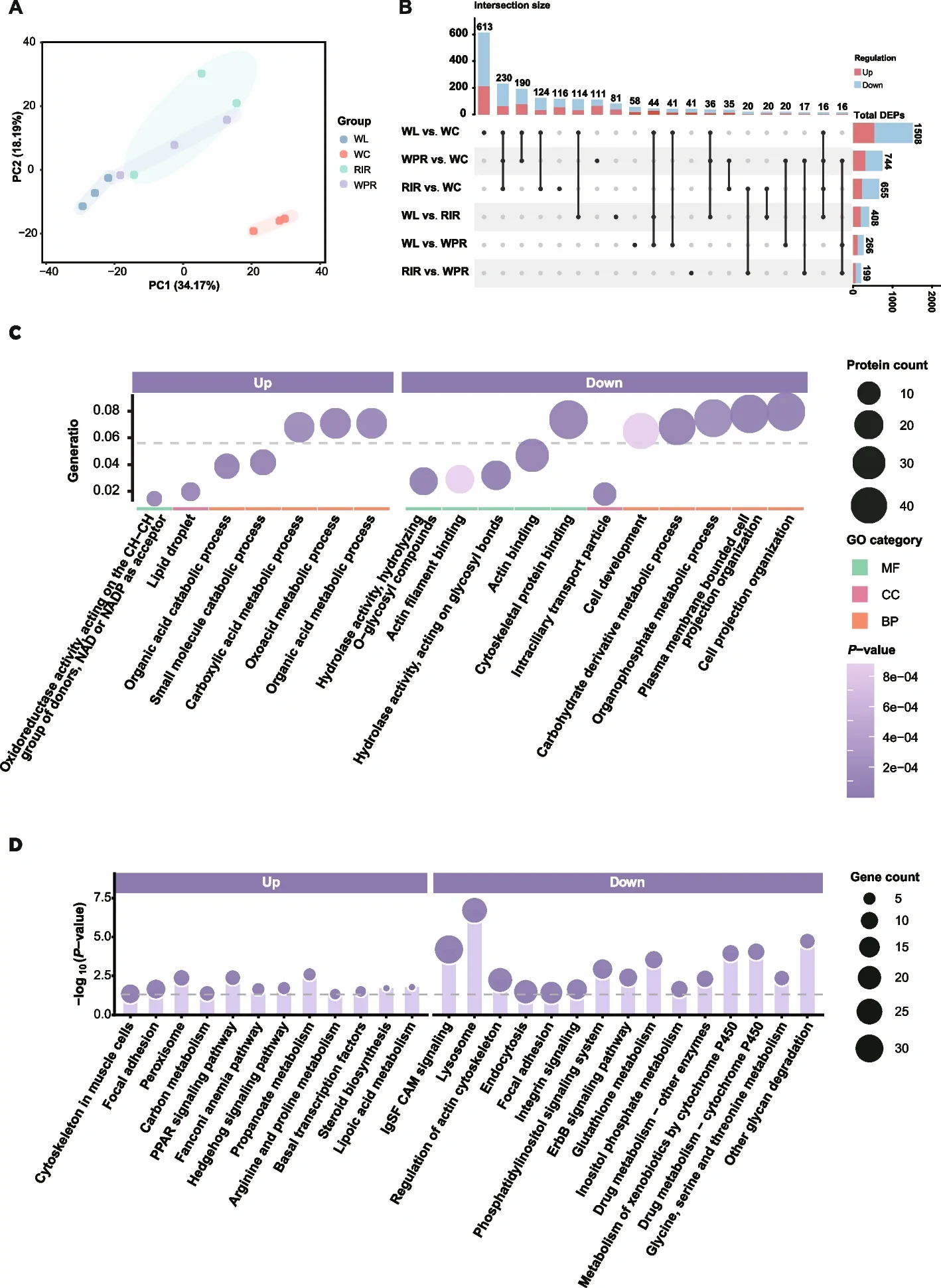
Proteomic profiling reveals differential protein expression and functional networks between WL and WC PGCs. **A** PCA score plot of the global proteomic profiles from the WL and WC groups. **B** UpSet plot illustrating the distribution of DEPs, filtered using the thresholds of |log_2_FC| ≥ 0.58 and *P* < 0.05. **C** GO enrichment analysis of the DEPs between WC and WL. The top 5 significantly enriched terms (*P* < 0.05) categorized by Biological Process (BP), Cellular Component (CC), and Molecular Function (MF), ranked by protein count, are displayed. **D** KEGG pathway enrichment analysis. The top 15 significantly enriched pathways (*P* < 0.05) for both upregulated and down-regulated DEPs are presented separately based on protein counts

Given that protein abundance acts as the direct executor of cellular phenotypes, this orthogonal approach provides a more definitive view of the intracellular landscape. In accordance with the overarching trends observed in the RNA-seq data, PCA highlighted the most robust inter-group divergence between WL and WC PGCs (Fig. 3A and Fig. S1B), indicating fundamentally distinct global protein expression patterns between the two breeds. We identified differentially expressed proteins (DEPs) the criteria of *P* < 0.05 and |Log_2_FC| ≥ 0.58 among the four breeds. The results (Fig. 3B) revealed that the WC versus WL comparison yielded the highest number of DEPs, comprising 561 upregulated and 1,014 downregulated targets. Notably, this specific comparison also identified 613 unique DEPs, a magnitude of breed-specific molecular divergence that far exceeds the differences observed between any other evaluated breed and the superior WL line, thereby quantitatively underscoring the severe intracellular reprogramming occurring in WC PGCs.

Aligning with the transcriptomic trends, GO enrichment of the DEPs emphasized massive shifts in “cytoskeletal organization” and “redox metabolism” (Fig. 3C). Downregulated DEPs were heavily concentrated in “actin binding”, “cytoskeletal protein binding”, and “cell projection organization”. This widespread suppression strongly signifies an extensive structural regression, which may disrupt normal cellular morphology and membrane integrity. Simultaneously, upregulated DEPs were highly enriched in “oxidoreductase activity, acting on the CH-CH group of donors, NAD or NADP as acceptor”, coupled with elevated organic/carboxylic acid metabolism and “lipid droplet”. These metabolic alterations are indicative of a profound cellular stress response and a rewiring of energy utilization. Concurrently, KEGG mapping at the proteomic level revealed a coordinated downregulation of the antioxidant synthesis network in WC PGCs. Specifically, a highly significant suppression of “Glutathione metabolism” was observed alongside the simultaneous inhibition of its upstream supplier, the “Glycine, serine and threonine metabolism” pathway. This dual suppression represents a severe biological bottleneck, as it ultimately cuts off the indispensable amino acid precursors for GSH biosynthesis [38–41]. Collectively, proteomic profiling indicates severe intracellular reprogramming in WC PGCs, where cytoskeletal abnormalities and a critical halt in glutathione metabolism dictate their deficient antioxidant defense.

### Integrated analysis identifies the *GSTA2 *and *CDK6* as candidate targets

**Fig. 4:**
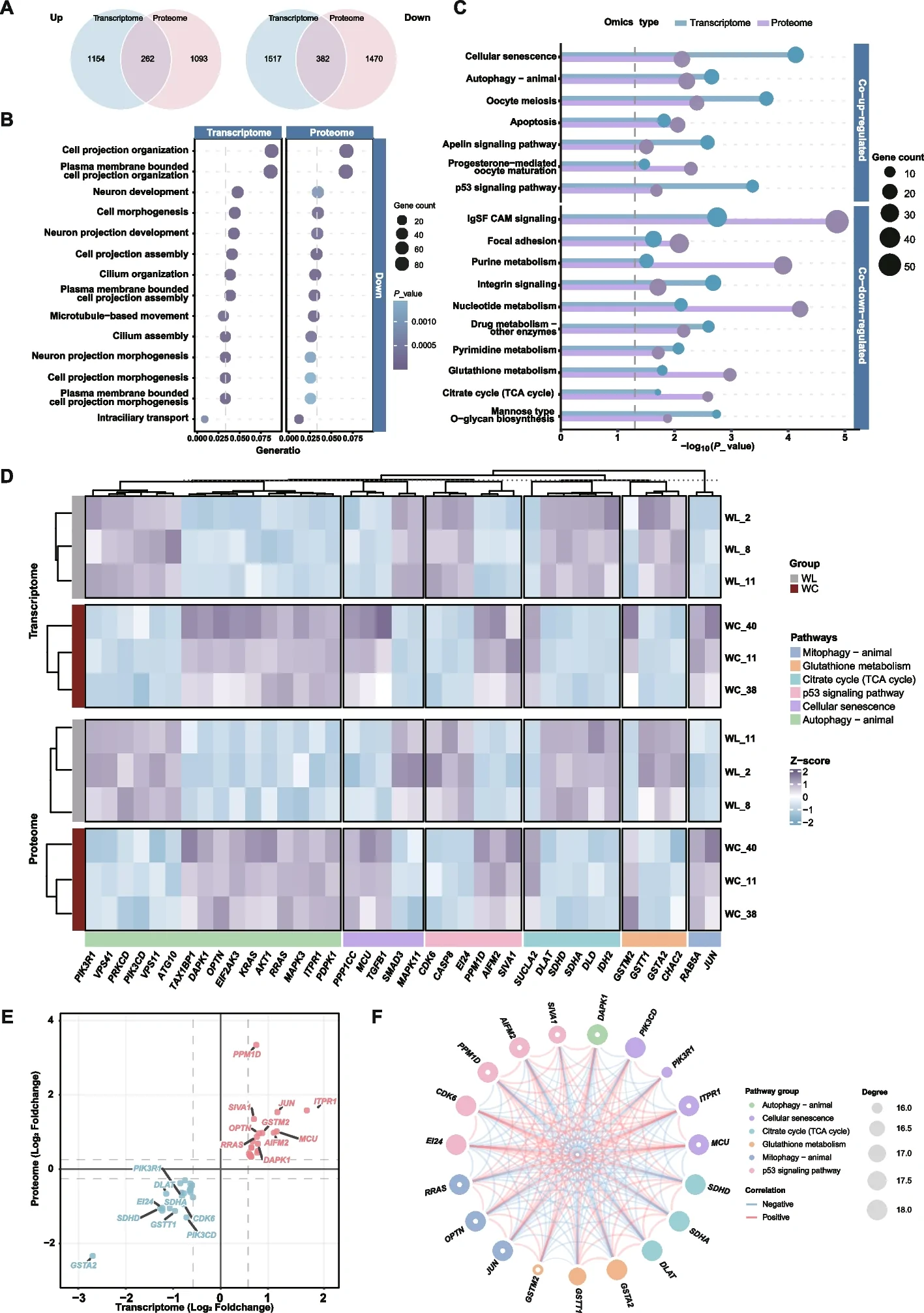
Integrated multi-omics analysis reveals the regulatory network driving the WC phenotype. **A** Venn diagrams of upregulated (left) and downregulated (right) DEGs between the transcriptome and proteome (|log_2_FC| ≥ 0.58, *P* < 0.05) and DEPs (|log_2_FC| ≥ 0.26, *P* < 0.05). **B** GO enrichment analysis of co-regulated targets. The top 15 significantly enriched BP terms for genes and proteins with consistent regulatory trends (co-upregulated or co-downregulated) are displayed. **C** KEGG pathway enrichment analysis. The top 10 significantly enriched pathways for both co-upregulated and co-downregulated targets are presented. **D** Heatmap illustrating the expression patterns of genes involved in key identified pathways, including Mitophagy–animal, Glutathione metabolism, Citrate cycle (TCA cycle), p53 signaling pathway, Cellular senescence, and Autophagy-animal. **E** Volcano plot highlighting the distribution and significance of genes associated with the aforementioned core pathways. **F** Co-expression network of top-ranked genes across key functional pathways. Node colors distinguish the six specific pathway groups. Node size is proportional to the topological degree of each gene within the network. Nodes marked with a white central dot indicate upregulated genes, whereas unmarked solid nodes represent downregulated genes. Pink and blue edges denote positive and negative co-expression correlations, respectively

To pinpoint the mechanistic effectors driving cell aggregation, we conducted an integrated transcriptomic and proteomic analysis. To capture functionally relevant candidate molecular shifts, we selected genes exhibiting concordant regulation across both levels, yielding 262 co-upregulated and 382 co-downregulated genes for downstream analysis (Fig. 4A). Consistent with our single-omics findings, the integrated multi-omics profiling (Fig. 4B and C) highlighted pronounced alterations in cell membrane dynamics, particularly a significant downregulation of “microtubule-based movement”, an event whose inhibition is known to promote cell aggregation. In addition, KEGG enrichment analysis revealed that upregulated DEGs and DEPs were significantly enriched in pathways related to cellular senescence and cell death, such as “Cellular senescence”, “Autophagy—animal”, “Apoptosis”, and the “p53 signaling pathway”. These results imply a potential activation of senescence and apoptotic signaling in WC PGCs. Meanwhile, the downregulated DEGs and DEPs aligned with the preceding results, exhibiting significant enrichment in “Glutathione metabolism” and the “Citrate cycle (TCA cycle)”. Thus, we propose that the pronounced suppression of glutathione metabolism, a key antioxidant hub, primarily accounts for the cell aggregation observed in WC PGCs. This impairment leads to the continuous accumulation of reactive oxygen species (ROS). Regarding cell fate, this oxidative overload broadly triggers p53-mediated senescence, autophagy, and underlying apoptotic programs, forcing the cells to adopt abnormal aggregation as a compensatory survival strategy.

Building upon these findings, we targeted the following specific pathways for subsequent analysis “Mitophagy—animal”, “p53 signaling pathway”, “Cellular senescence”, “Autophagy—animal”, “Glutathione metabolism”, and the “Citrate cycle (TCA cycle)”. Initially, we generated a heatmap for DEGs and DEPs that exhibited consistent regulatory trends across the transcriptomic and proteomic datasets (Fig. 4D). This visualization revealed that the targets identified within the glutathione and TCA pathways were almost universally downregulated. Subsequent volcano plot analysis of these targets clearly demonstrated that *GSTA2* was significantly downregulated at both the transcriptomic and proteomic levels. Concurrently, *CDK6*, a gene intricately involved in cell cycle progression, was also significantly suppressed (Fig. 4E). Next, we constructed a co-expression network utilizing the top-ranked genes from six relevant pathways (Fig. 4F). This network reveals complex interactions among these hub genes, tightly linking key biological processes such as cellular antioxidant defense and apoptosis. Ultimately, this intricate molecular crosstalk demonstrates that the aberrant phenotype observed in WC cells is the result of the synergistic action of multiple interconnected pathways. Overall, multi-omics integration demonstrates that the reduced antioxidant defense and slow proliferation of WC PGCs stem mechanistically from a significant blockade in glutathione metabolism coupled with elevated p53 signaling. Notably, the significant downregulation of *GSTA2* and *CDK6* appears to play an important role in this process.

### Oxidative stress validation and antioxidant selection in WC PGCs

**Fig. 5:**
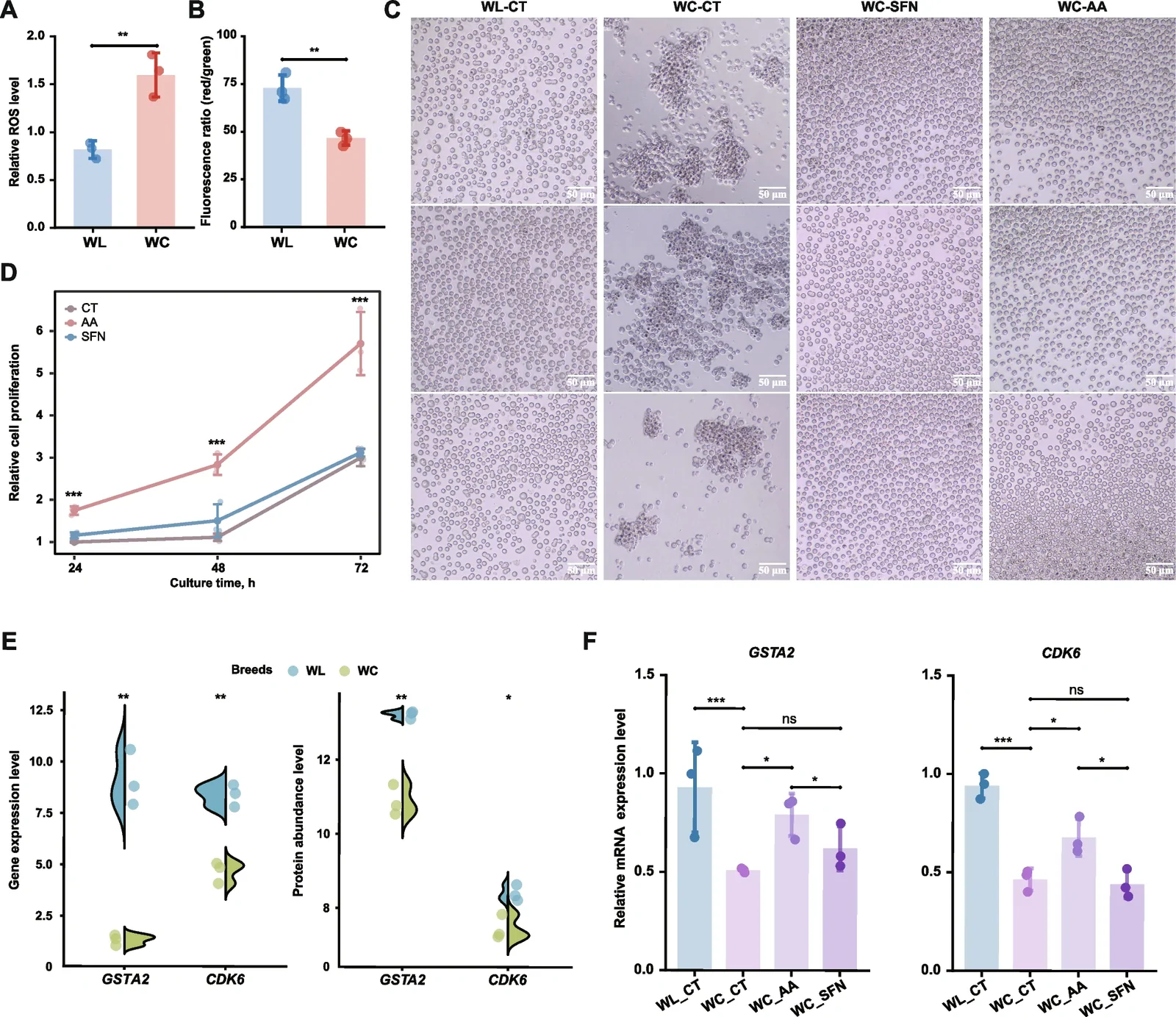
Oxidative stress validation and antioxidant optimization to rescue aggregation and proliferation in WC PGCs.** A** Intracellular ROS levels in WL and WC PGCs, quantified utilizing the DCFH-DA fluorescent probe. **B** Basal lipid peroxidation levels, assessed via the BODIPY 581/591 C11 probe and expressed as the Red/Green fluorescence intensity ratio. **C** Representative morphological images of WC PGCs (seeded at 6 × 10^4^ cells/well in 24-well plates) following a 48-h treatment with SFN and AA. Scale bar = 50 μm. **D** Cell proliferation post-intervention, assessed via the CCK-8 assay. Cells were seeded in 96-well plates at 4 × 10^3^ cells/well. Absorbance was recorded at 24, 48, and 72 h, and data were normalized to the 24 h baseline to reflect dynamic proliferation rates. **E** Expression value of *GSTA2* and *CDK6* in the transcriptome (FPKM) and proteome (protein abundance) of WL and WC PGCs. **F** Relative mRNA expression levels of *GSTA2* and *CDK6* post 48-h treatment, quantified by RT-qPCR. All data are presented as the mean ± SD (*n* = 3 independent biological replicates). Statistical significance was evaluated using *t*-test (^*^P < 0.05, ^**^P < 0.01, and ^***^P < 0.001 compared with the control)

Lipid peroxidation, a direct consequence of oxidative membrane damage, is a key indicator of redox stress. WC PGCs exhibited significantly higher intracellular ROS (*P* < 0.01) and lipid peroxidation (*P* < 0.05) compared to WL PGCs (Fig. 5A and B), confirming their inherently weak antioxidant capacity and vulnerability to culture-induced oxidative stress, as predicted by multi-omics analyses. To rescue this phenotype, we screened three antioxidants: L-sulforaphane (SFN), GSH-EE, and AA. All three reduced ROS levels in WC PGCs; however, GSH-EE increasingly suppressed proliferation at higher concentrations, whereas SFN and AA exhibited minimal cytotoxicity. We therefore selected SFN (1 μmol/L) and AA (0.5 mmol/L) for subsequent experiments (Fig. S2A–C). Light microscopy revealed that both agents ameliorated the aberrant cell aggregation of WC PGCs (Fig. 5C), supporting oxidative stress as a primary contributor of this phenotype. In CCK-8 proliferation assays, SFN led to a slight increase in proliferation, whereas AA significantly accelerated it (*P* < 0.001), restoring the growth trajectory to a level comparable to that of WL PGCs (Fig. 5D). Multi-omics data had shown lower expression of *GSTA2* and *CDK6* in WC PGCs at both transcriptome and proteome levels (Fig. 5E). Functionally, *GSTA2* mitigates oxidative damage by detoxifying reactive byproducts [42], whereas *CDK6* drives the G1-to-S transition [43]. RT-qPCR analysis indicated that both SFN and AA upregulated *GSTA2* mRNA, with AA inducing a substantially greater increase; AA also upregulated *CDK6* expression (Fig. 5F). Thus, both agents enhance antioxidant defense via *GSTA2*, reducing ROS and mitigating aggregation, while AA additionally promotes proliferation through *CDK6*. These findings demonstrate that effective ROS neutralization alleviates abnormal aggregation and accelerates cell proliferation.

### Ascorbic acid promotes PGCs proliferation and alleviates oxidative stress

**Fig. 6:**
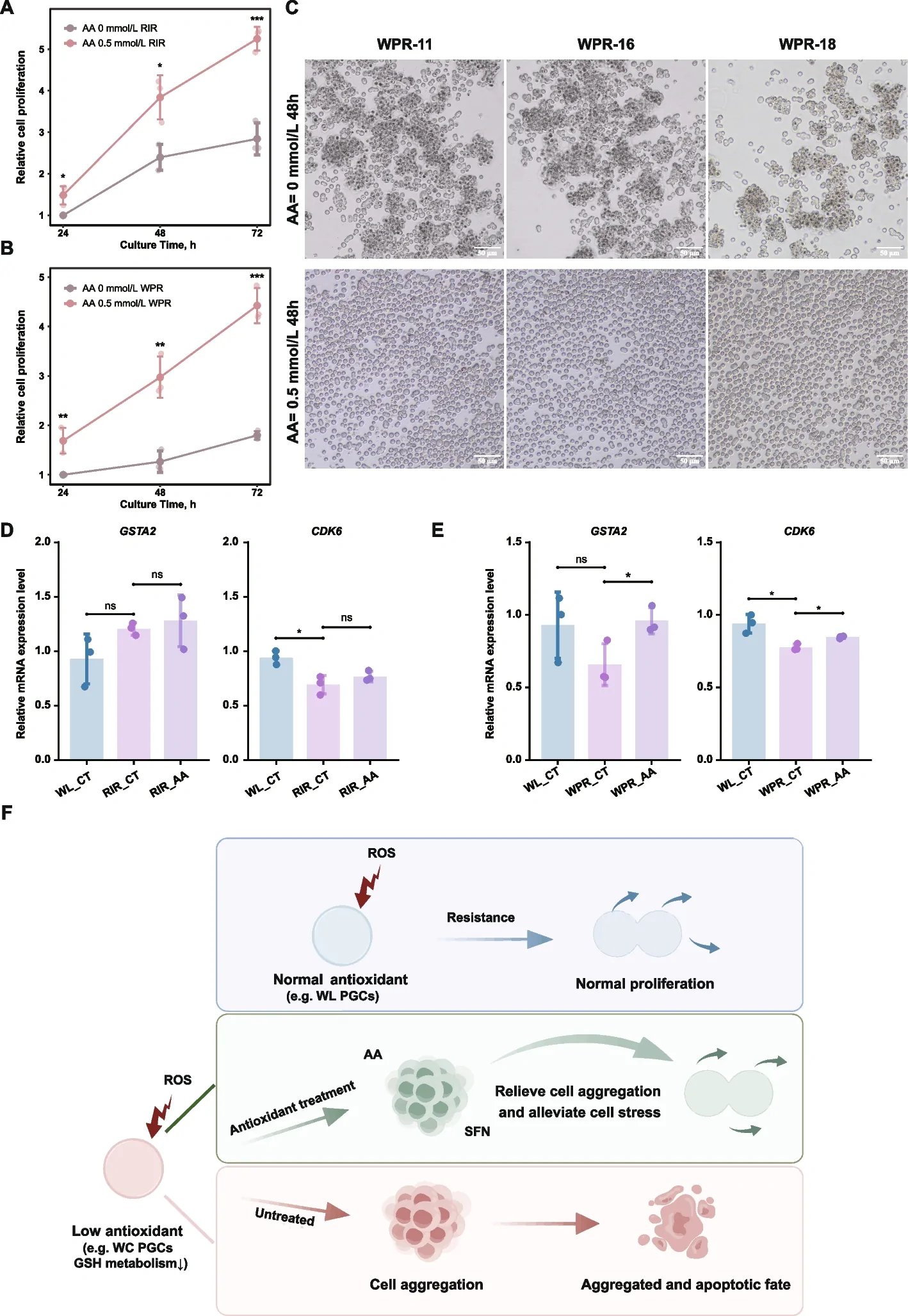
Ascorbic acid promotes proliferation and alleviates aggregation in PGCs across breeds via *GSTA2* and *CDK6* upregulation. **A** CCK-8 assay measuring proliferation of RIR PGCs treated with AA. **B** CCK-8 assay measuring proliferation of WPR PGCs treated with AA. **C** Light microscopy images of WPR PGCs after 48 h of AA treatment, showing morphological improvement. **D** RT-qPCR analysis of *GSTA2* and *CDK6* mRNA levels in RIR PGCs following 48 h of AA treatment. **E** RT-qPCR analysis of *GSTA2* and *CDK6* mRNA levels in WPR PGCs following 48 h of AA treatment. **F** Schematic model illustrating how antioxidant supplementation enhances cell proliferation and alleviates cell aggregation. ^*^P < 0.05, ^**^P < 0.01, ^***^P < 0.001

To determine whether 0.5 mmol/L AA exerts a consistent effect on PGCs from different chicken breeds, we further examined the RIR and WPR breeds. Consistent with the results obtained in the WC breed, AA treatment increased the proliferation rate of PGCs in both RIR (Fig. 6A) and WPR (Fig. 6B), with a more pronounced effect observed in WPR PGCs. Furthermore, the clustering phenotype of WPR cells was ameliorated (Fig. 6C). RT-qPCR analysis revealed that AA supplementation upregulated the mRNA expression levels of *GSTA2* and *CDK6* in RIR PGCs (Fig. 6D), but these changes were not statistically significant compared to the untreated control. In contrast, the expression of both genes was significantly increased in WPR PGCs (Fig. 6E). These results suggest that AA may exert a more pronounced ameliorative effect on PGCs with slower proliferation rates and a suboptimal cellular state.

## Discussion

In the present study, we systematically compared the proliferation and morphological characteristics of PGCs from different chicken breeds during in vitro culture. Among the four breeds, the WL breed outperformed the other breeds in terms of both line establishment efficiency and derivation time. Conversely, the WC and WPR PGCs were highly susceptible to extensive cell aggregation.

Previous studies have demonstrated that oxidative stress induces apoptosis and cell aggregation. The downregulation of the TCA cycle typically indicates impaired mitochondrial metabolic activity [44], which is frequently accompanied by excessive ROS accumulation [45, 46] and a detrimental feedback loop that further compromises mitochondrial function [47]. Under high oxidative stress, cells downregulate aerobic metabolism to limit further ROS production [28, 48] and alter adhesion molecule expression to promote aggregation [38]. Consistent with these findings, our integrated multiomics analysis revealed that WC PGCs were under pronounced oxidative stress relative to WL cells: glutathione metabolism and the TCA cycle were significantly downregulated, while pathways linked to cellular senescence and apoptosis were upregulated. Additionally, genes downregulated in both omics datasets were enriched in “microtubule-based movement”, and the inhibition of microtubule synthesis has been associated with enhanced cell adhesion [49, 50]. Subsequent assays confirmed that the intracellular ROS and lipid peroxidation levels in the WC PGCs were significantly greater than those in the WL PGCs, indicating compromised resistance to the inevitable oxidative stress encountered in vitro.

To increase antioxidant capacity, we employed three targeted interventions: activating the Nrf2 pathway, enhancing GSH signaling, and providing general antioxidant supplementation. Previous studies have indicated that the Nrf2 pathway is a master regulator of antioxidant defenses [51], and its inhibition accelerates apoptosis in chicken PGCs [28]. Thus, we utilized SFN, a potent Nrf2 activator [52, 53]. To target the GSH pathway, we supplemented GSH-EE, a cell-permeable substrate, to directly increase the intracellular GSH pool and bypass metabolic bottlenecks [54, 55]. Additionally, we applied AA, a ubiquitous antioxidant that directly neutralizes ROS and is known to accelerate cell proliferation [56, 57]. Drawing upon previous literature, we evaluated SFN and AA across concentration ranges of 0–5 μmol/L and 0–10 mmol/L, respectively. Our results demonstrated that treatment with 1 μmol/L SFN or 0.5 mmol/L AA for 48 h effectively reduced intracellular ROS levels without causing cytotoxicity. Notably, AA supplementation significantly accelerated the proliferation rate of WC PGCs. To understand the molecular basis underlying this proliferative effect, we next examined whether AA treatment altered the expression of genes implicated in oxidative stress response and cell cycle progression.

Leveraging these integrated multiomics insights, we identified *GSTA2* and *CDK6* as candidate genes. Specifically, *GSTA2* belongs to the glutathione S-transferase (GST) family. As previously demonstrated by Rao and Shaha [58], GSTs represent an essential component of the adaptive response to oxidative stress in germ cells, playing a crucial role in detoxifying lipid peroxidation products. Furthermore, Zhang et al. [59] reported that *CDK6* facilitates the rapid entry of embryonic stem cells into the S phase. Importantly, RT-qPCR analysis revealed that the mRNA expression of both *GSTA2* and *CDK6* was significantly upregulated following AA treatment, thereby providing a mechanistic basis for the accelerated proliferation observed in AA-treated WC PGCs. Notably, we extended these findings to RIR and WPR breeds. AA consistently promoted PGC proliferation across breeds, with the most pronounced rescue in WPR cells, which exhibited slower basal proliferation and a more compromised cellular state. In these WPR PGCs, AA also significantly upregulated *GSTA2* and *CDK6* expression, mirroring the molecular changes observed in WC cells. These results suggest that AA has a greater beneficial effect on PGCs with intrinsically weaker antioxidant capacity.

On the basis of these findings, we propose the following overarching mechanistic model to elucidate breed-specific PGC dynamics. Under in vitro culture conditions, the spontaneous and continuous generation of ROS is a universally unavoidable phenomenon [60]. Cells with robust antioxidant defenses (e.g., WL PGCs) readily maintain redox homeostasis. whereas those with inherently weak antioxidant capacity, such as WC PGCs, are highly susceptible to oxidative stress, which suppresses cell cycle progression and triggers aberrant intercellular aggregation. We demonstrated that supplementation with exogenous antioxidants, particularly AA, effectively compensates for this deficiency. By scavenging excessive ROS, AA alleviates oxidative damage, successfully ameliorates cell aggregation, and actively accelerates proliferation (Fig. 6F).

Therefore, our findings offer a potential strategy for optimizing chicken PGC culture, and this antioxidant-based approach may be applicable to PGCs from other breeds. We demonstrated that ROS accumulation contributes, at least in part, to PGC aggregation, thus providing a useful reference for culture optimization. For PGC lines with severe aggregation, measuring intracellular ROS or expression of candidate genes (e.g., *GSTA2* and *CDK6*) could help assess oxidative stress and guide targeted supplementation with AA or other antioxidants. However, the precise mechanisms underlying PGC aggregation remain to be fully deciphered, and future studies involving functional validation, such as gene knockdown or overexpression, are required to establish direct causality. Additionally, while short-term AA supplementation proved beneficial, whether its continuous application during long-term culture might induce adverse effects, such as compromising genomic stability or altering differentiation potential, warrants further investigation. Moreover, systematic data on the impact of AA on the derivation efficiency of chicken PGC lines are currently lacking, and this also represents an important direction for future research. Finally, while we confirmed clear phenotypic and antioxidant disparities between WC and WL PGCs, whether these differences generally extend to broiler and layer breeds remains to be investigated.

## Conclusions

In conclusion, our integrated multi‑omics analysis indicated that dysregulation of GSH metabolism and p53 signaling, together with aberrant expression of *GSTA2* and *CDK6*, predisposes WC PGCs to oxidative stress and ROS accumulation, which in turn promotes cell aggregation. Among the tested pharmacological interventions, AA effectively upregulated both *GSTA2* and *CDK6* expression, thereby ameliorating spontaneous cell aggregation and significantly accelerating proliferation in WC PGCs. Importantly, we extended these findings to RIR and WPR breeds, where AA consistently promoted PGC proliferation, with the most pronounced rescue observed in WPR cells that exhibit slower proliferation and a more compromised cellular state. In WPR PGCs, AA significantly upregulated *GSTA2* and *CDK6*, mirroring the molecular changes seen in WC cells. Collectively, these results demonstrate that AA exerts a broadly beneficial effect on PGCs with compromised antioxidant capacity, restoring redox balance and cell cycle progression across breeds.

## Supplementary Information

Additional file 1: Fig. S1. Number of DEGs and proteins DEPs identified across the four breeds. Fig. S2. Dose-response assessment of three antioxidant interventions on PGC viability.

Additional file 2: Table S1. Efficiency of PGC line establishment across different chicken breeds. Table S2. Raw gene count matrix from the transcriptomic analysis. Table S3. Raw protein expression abundances following seqKNN imputation. Table S4. Primer sequences used for RT-qPCR experiment.

Additional file 3: Uncropped gel image corresponding to Fig.1.

## Data Availability

The datasets supporting the conclusions of this article are included within the article and its additional files.

## Abbreviations

AA: Ascorbic acid
GSH: Glutathione
PGCs: Primordial germ cells
RIR: Rhode Island Red
ROS: Reactive oxygen species
SFN: L-Sulforaphane
WC: White Cornish
WL: White Leghorn
WPR: White Plymouth Rock

## References

[1] Stern CD. The chick; a great model system becomes even greater. Dev Cell. 2005;8(1):9–17. 10.1016/j.devcel.2004.11.018. 15621526

[2] Cogburn LA, Wang X, Carre W, Rejto L, Porter TE, Aggrey SE, et al. Systems-wide chicken DNA microarrays, gene expression profiling, and discovery of functional genes. Poult Sci. 2003;82(6):939–51. 10.1093/ps/82.6.939. 12817449

[3] Thornton PK. Livestock production: recent trends, future prospects. Philos Trans R Soc Lond B Biol Sci. 2010;365(1554):2853–67. 10.1098/rstb.2010.0134. PMC2935116 20713389

[4] Han JY, Lee HG, Park YH, Hwang YS, Kim SK, Rengaraj D, et al. Acquisition of pluripotency in the chick embryo occurs during intrauterine embryonic development via a unique transcriptional network. J Anim Sci Biotechnol. 2018;9:31. 10.1186/s40104-018-0246-0. PMC5891889 29644074

[5] Henchion M, Moloney AP, Hyland J, Zimmermann J, McCarthy S. Review: Trends for meat, milk and egg consumption for the next decades and the role played by livestock systems in the global production of proteins. Animal. 2021;15(Suppl 1):100287. 10.1016/j.animal.2021.100287. 34312092

[6] Fulton JE. Avian genetic stock preservation: an industry perspective. Poult Sci. 2006;85(2):227–31. 10.1093/ps/85.2.227. 16523618

[7] Lyall J, Irvine RM, Sherman A, McKinley TJ, Nunez A, Purdie A, et al. Suppression of avian influenza transmission in genetically modified chickens. Science. 2011;331(6014):223–6. 10.1126/science.1198020. 21233391

[8] Idoko-Akoh A, Goldhill DH, Sheppard CM, Bialy D, Quantrill JL, Sukhova K, et al. Creating resistance to avian influenza infection through genome editing of the ANP32 gene family. Nat Commun. 2023;14:6136. 10.1038/s41467-023-41476-3. PMC10564915 37816720

[9] Muir WM, Wong GK, Zhang Y, Wang J, Groenen MA, Crooijmans RP, et al. Genome-wide assessment of worldwide chicken SNP genetic diversity indicates significant absence of rare alleles in commercial breeds. Proc Natl Acad Sci U S A. 2008;105(45):17312–7. 10.1073/pnas.0806569105. PMC2582254 18981413

[10] Sun Y, Li Y, Zong Y, Mehaisen GMK, Chen J. Poultry genetic heritage cryopreservation and reconstruction: advancement and future challenges. J Anim Sci Biotechnol. 2022;13:115. 10.1186/s40104-022-00768-2. PMC9549680 36210477

[11] van de Lavoir MC, Diamond JH, Leighton PA, Mather-Love C, Heyer BS, Bradshaw R, et al. Germline transmission of genetically modified primordial germ cells. Nature. 2006;441(7094):766–9. 10.1038/nature04831. 16760981

[12] Woodcock ME, Gheyas AA, Mason AS, Nandi S, Taylor L, Sherman A, et al. Reviving rare chicken breeds using genetically engineered sterility in surrogate host birds. Proc Natl Acad Sci U S A. 2019;116(42):20930–7. 10.1073/pnas.1906316116. PMC6800374 31575742

[13] Kinoshita K, Tanabe K, Nakamura Y, Nishijima KI, Suzuki T, Okuzaki Y, et al. PGC-based cryobanking, regeneration through germline chimera mating, and CRISPR/Cas9-mediated *TYRP1* modification in indigenous Chinese chickens. Commun Biol. 2024;7:1127. 10.1038/s42003-024-06775-5. PMC11399235 39271811

[14] Kuwana T. Migration of avian primordial germ cells toward the gonadal anlage: (avian/PGC/migration/presumptive gonad/chimera). Dev Growth Differ. 1993;35(3):237–43. 10.1111/j.1440-169X.1993.00237.x. 37281712

[15] Stebler J, Spieler D, Slanchev K, Molyneaux KA, Richter U, Cojocaru V, et al. Primordial germ cell migration in the chick and mouse embryo: the role of the chemokine SDF-1/CXCL12. Dev Biol. 2004;272(2):351–61. 10.1016/j.ydbio.2004.05.009. 15282153

[16] Kim YM, Han JY. The early development of germ cells in chicken. Int J Dev Biol. 2018;62:145–52. 10.1387/ijdb.170283jh. 29616722

[17] Tsunekawa N, Naito M, Sakai Y, Nishida T, Noce T. Isolation of chicken vasa homolog gene and tracing the origin of primordial germ cells. Development. 2000;127(12):2741–50. 10.1242/dev.127.12.2741. 10821771

[18] Kang KS, Lee HC, Kim HJ, Lee HG, Kim YM, Lee HJ, et al. Spatial and temporal action of chicken primordial germ cells during initial migration. Reprod. 2015;149(2):179–87. 10.1530/REP-14-0433. 25550524

[19] Choi JW, Kim S, Kim TM, Kim YM, Seo HW, Park TS, et al. Basic fibroblast growth factor activates MEK/ERK cell signaling pathway and stimulates the proliferation of chicken primordial germ cells. PLoS ONE. 2010;5(9):e12968. 10.1371/journal.pone.0012968. PMC2944891 20886037

[20] Macdonald J, Taylor L, Sherman A, Kawakami K, Takahashi Y, Sang HM, et al. Efficient genetic modification and germ-line transmission of primordial germ cells using piggyBac and Tol2 transposons. Proc Natl Acad Sci U S A. 2012;109(23):E1466–72. 10.1073/pnas.1118715109. PMC3384192 22586100

[21] Han Y, Kwon CY, Han JY. Improvement of chicken genome editing efficiency in vitro using ribonucleoprotein-mediated CRISPR/Cas9 delivery. J Anim Sci Biotechnol. 2026;17:82. 10.1186/s40104-026-01402-1. PMC13137644 42071241

[22] Park TS, Han JY. *piggyBac* transposition into primordial germ cells is an efficient tool for transgenesis in chickens. Proc Natl Acad Sci U S A. 2012;109(24):9337–41. 10.1073/pnas.1203823109. PMC3386051 22645326

[23] Oishi I, Yoshii K, Miyahara D, Kagami H, Tagami T. Targeted mutagenesis in chicken using CRISPR/Cas9 system. Sci Rep. 2016;6:23980. 10.1038/srep23980. PMC4822141 27050479

[24] Lee HJ, Yoon JW, Jung KM, Kim YM, Park JS, Lee KY, et al. Targeted gene insertion into Z chromosome of chicken primordial germ cells for avian sexing model development. FASEB J. 2019;33(7):8519–29. 10.1096/fj.201802671R. 30951374

[25] Kim GD, Lee JH, Song S, Kim SW, Han JS, Shin SP, et al. Generation of myostatin-knockout chickens mediated by D10A-Cas9 nickase. FASEB J. 2020;34(4):5688–96. 10.1096/fj.201903035R. 32100378

[26] Naito M, Harumi T, Kuwana T. Long-term culture of chicken primordial germ cells isolated from embryonic blood and production of germline chimaeric chickens. Anim Reprod Sci. 2015;153:50–61. 10.1016/j.anireprosci.2014.12.003. 25578502

[27] Whyte J, Glover JD, Woodcock M, Brzeszczynska J, Taylor L, Sherman A, et al. FGF, Insulin, and SMAD signaling cooperate for avian primordial germ cell self-renewal. Stem Cell Reports. 2015;5(6):1171–82. 10.1016/j.stemcr.2015.10.008. PMC4682126 26677769

[28] Niu YJ, Yuan G, Ren W, Wu J, Liu G, Zou M, et al. NRF2 deficiency impairs proliferation and survival of chicken primordial germ cells via oxidative stress, mitochondrial dysfunction and apoptosis. Poult Sci. 2026;105(6):106765. 10.1016/j.psj.2026.106765. PMC13011206 41850075

[29] Zampiga M, Flees J, Meluzzi A, Dridi S, Sirri F. Application of omics technologies for a deeper insight into quali-quantitative production traits in broiler chickens: A review. J Anim Sci Biotechnol. 2018;9:61. 10.1186/s40104-018-0278-5. PMC6130060 30214720

[30] Schieber M, Chandel NS. ROS function in redox signaling and oxidative stress. Curr Biol. 2014;24(10):R453–62. 10.1016/j.cub.2014.03.034. PMC4055301 24845678

[31] Ichikawa K, McGrew MJ. Transcriptome profiles for defining avian primordial germ cell development. Protein Expr Purif. 2026;237:106812. 10.1016/j.pep.2025.106812. 40945630

[32] Han JY, Park YH. Primordial germ cell-mediated transgenesis and genome editing in birds. J Anim Sci Biotechnol. 2018;9:19. 10.1186/s40104-018-0234-4. PMC5791193 29423217

[33] Chen S. Ultrafast one-pass FASTQ data preprocessing, quality control, and deduplication using fastp. Imeta. 2023;2(2):e107. 10.1002/imt2.107. PMC10989850 38868435

[34] Kim D, Paggi JM, Park C, Bennett C, Salzberg SL. Graph-based genome alignment and genotyping with HISAT2 and HISAT-genotype. Nat Biotechnol. 2019;37(8):907–15. 10.1038/s41587-019-0201-4. PMC7605509 31375807

[35] Love MI, Huber W, Anders S. Moderated estimation of fold change and dispersion for RNA-seq data with DESeq2. Genome Biol. 2014;15(12):550. 10.1186/s13059-014-0550-8. PMC4302049 25516281

[36] Yu G. Thirteen years of clusterProfiler. Innovation (Camb). 2024;5(6):100722. 10.1016/j.xinn.2024.100722. PMC11551487 39529960

[37] Wickham H. Elegant graphics for data analysis. Cham: Springer; 2016.

[38] Piskounova E, Agathocleous M, Murphy MM, Hu Z, Huddlestun SE, Zhao Z, et al. Oxidative stress inhibits distant metastasis by human melanoma cells. Nature. 2015;527(7577):186–91. 10.1038/nature15726. PMC4644103 26466563

[39] Bailey AP, Koster G, Guillermier C, Hirst EM, MacRae JI, Lechene CP, et al. Antioxidant role for lipid droplets in a stem cell niche of drosophila. Cell. 2015;163(2):340–53. 10.1016/j.cell.2015.09.020. PMC4601084 26451484

[40] Locasale JW. Serine, glycine and one-carbon units: cancer metabolism in full circle. Nat Rev Cancer. 2013;13(8):572–83. 10.1038/nrc3557. PMC3806315 23822983

[41] Harris IS, Treloar AE, Inoue S, Sasaki M, Gorrini C, Lee KC, et al. Glutathione and thioredoxin antioxidant pathways synergize to drive cancer initiation and progression. Cancer Cell. 2015;27(2):211–22. 10.1016/j.ccell.2014.11.019. 25620030

[42] Hayes JD, Flanagan JU, Jowsey IR. Glutathione transferases. Annu Rev Pharmacol Toxicol. 2005;45(1):51–88. 10.1146/annurev.pharmtox.45.120403.095857. 15822171

[43] Malumbres M, Barbacid M. Cell cycle, CDKs and cancer: a changing paradigm. Nat Rev Cancer. 2009;9(3):153–66. 10.1038/nrc2602. 19238148

[44] Martinez-Reyes I, Chandel NS. Mitochondrial TCA cycle metabolites control physiology and disease. Nat Commun. 2020;11:102. 10.1038/s41467-019-13668-3. PMC6941980 31900386

[45] Zorov DB, Juhaszova M, Sollott SJ. Mitochondrial reactive oxygen species (ROS) and ROS-induced ROS release. Physiol Rev. 2014;94(3):909–50. 10.1152/physrev.00026.2013. PMC4101632 24987008

[46] Murphy MP. How mitochondria produce reactive oxygen species. Biochem J. 2009;417(1):1–13. 10.1042/BJ20081386. PMC2605959 19061483

[47] Tretter L, Adam-Vizi V. Inhibition of Krebs cycle enzymes by hydrogen peroxide: A key role of α-ketoglutarate dehydrogenase in limiting NADH production under oxidative stress. J Neurosci. 2000;20(24):8972–9. 10.1523/JNEUROSCI.20-24-08972.2000. PMC6773008 11124972

[48] Gorlach A, Bertram K, Hudecova S, Krizanova O. Calcium and ROS: a mutual interplay. Redox Biol. 2015;6:260–71. 10.1016/j.redox.2015.08.010. PMC4556774 26296072

[49] Enomoto T. Microtubule disruption induces the formation of actin stress fibers and focal adhesions in cultured cells: possible involvement of the rho signal cascade. Cell Struct Funct. 1996;21(5):317–26. 10.1247/csf.21.317. 9118237

[50] Ng DH, Humphries JD, Byron A, Millon-Fremillon A, Humphries MJ. Microtubule-dependent modulation of adhesion complex composition. PLoS ONE. 2014;9(12):e115213. 10.1371/journal.pone.0115213. PMC4272306 25526367

[51] Dinkova-Kostova AT, Fahey JW, Kostov RV, Kensler TW. KEAP1 and done? Targeting the NRF2 pathway with sulforaphane. Trends Food Sci Technol. 2017;69(Pt B):257–69. 10.1016/j.tifs.2017.02.002. PMC5725197 29242678

[52] Jaiswal AK. Nrf2 signaling in coordinated activation of antioxidant gene expression. Free Radic Biol Med. 2004;36(10):1199–207. 10.1016/j.freeradbiomed.2004.02.074. 15110384

[53] Houghton CA, Fassett RG, Coombes JS. Sulforaphane and other nutrigenomic Nrf2 activators: can the clinician’s expectation be matched by the reality? Oxid Med Cell Longev. 2016;2016:7857186. 10.1155/2016/7857186. PMC4736808 26881038

[54] Anderson ME, Powrie F, Puri RN, Meister A. Glutathione monoethyl ester: preparation, uptake by tissues, and conversion to glutathione. Arch Biochem Biophys. 1985;239(2):538–48. 10.1016/0003-9861(85)90723-4. 4004275

[55] Zeevalk GD, Manzino L, Sonsalla PK, Bernard LP. Characterization of intracellular elevation of glutathione (GSH) with glutathione monoethyl ester and GSH in brain and neuronal cultures: relevance to Parkinson’s disease. Exp Neurol. 2007;203(2):512–20. 10.1016/j.expneurol.2006.09.004. PMC1839874 17049515

[56] D’Aniello C, Cermola F, Patriarca EJ, Minchiotti G. Vitamin C in stem cell biology: impact on extracellular matrix homeostasis and epigenetics. Stem Cells Int. 2017;2017:8936156. 10.1155/2017/8936156. PMC5415867 28512473

[57] Esteban MA, Wang T, Qin B, Yang J, Qin D, Cai J, et al. Vitamin C enhances the generation of mouse and human induced pluripotent stem cells. Cell Stem Cell. 2010;6(1):71–9. 10.1016/j.stem.2009.12.001. 20036631

[58] Rao AV, Shaha C. Role of glutathione S-transferases in oxidative stress-induced male germ cell apoptosis. Free Radic Biol Med. 2000;29(10):1015–27. 10.1016/s0891-5849(00)00408-1. 11084290

[59] Zhang X, Neganova I, Przyborski S, Yang C, Cooke M, Atkinson SP, et al. A role for NANOG in G1 to S transition in human embryonic stem cells through direct binding of CDK6 and CDC25A. J Cell Biol. 2009;184(1):67–82. 10.1083/jcb.200801009. PMC2615089 19139263

[60] Halliwell B. Cell culture, oxidative stress, and antioxidants: avoiding pitfalls. Biomed J. 2014;37(3):99–105. 10.4103/2319-4170. 24923566

