# Supplementary material for: Molecular insights into ROS-induced aggregation in chicken primordial germ cells and its alleviation by ascorbic acid
Source: J Anim Sci Biotechnol. 2026 Nov 9;17:204. doi: 10.1186/s40104-026-01521-9 (PMC13746570; doi:10.1186/s40104-026-01521-9)
Supplement: Supplementary file 1 [file 40104_2026_1521_MOESM3_ESM.pdf]

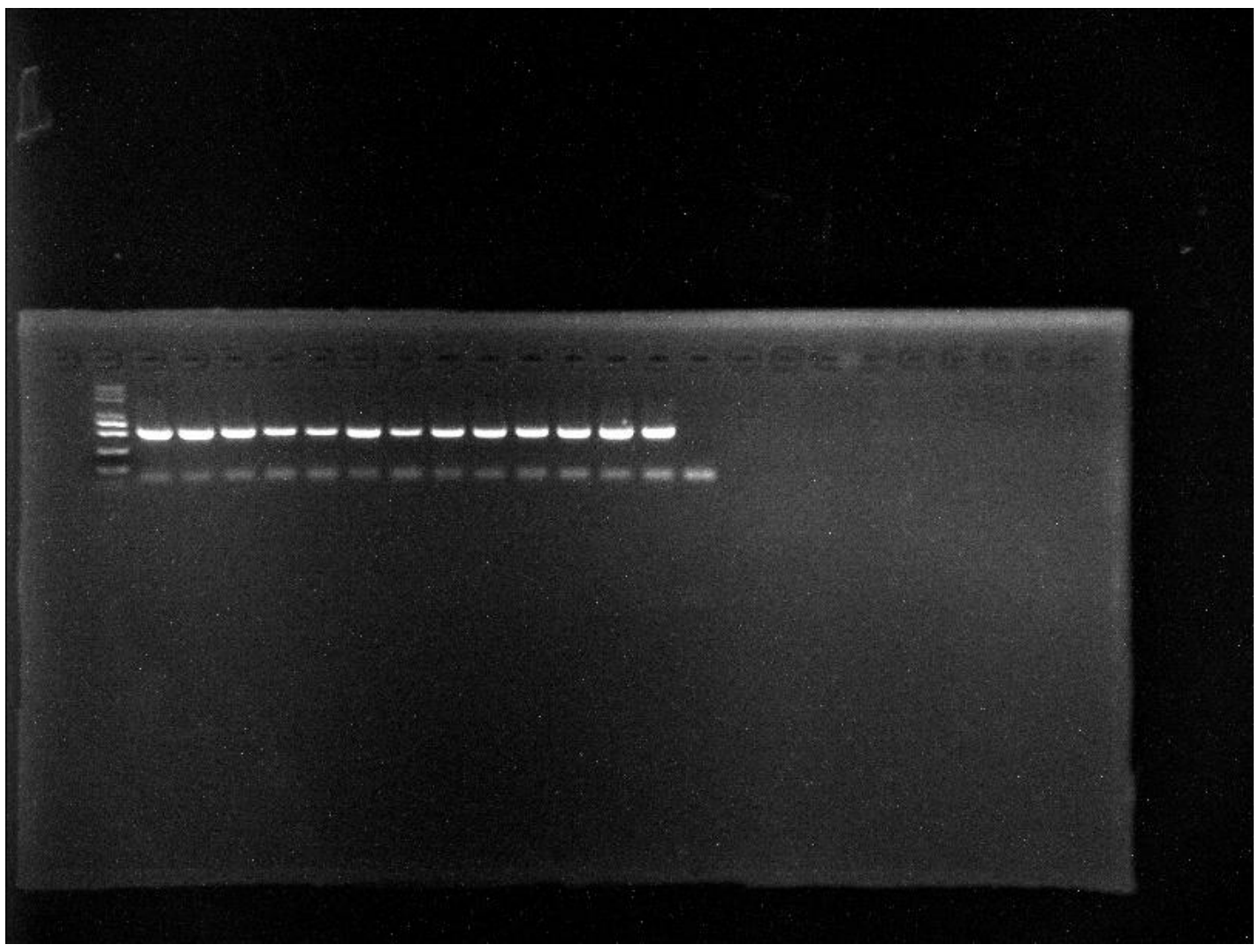

Fig. S3 Uncropped gel image corresponding to Fig.1.
