# Supplementary material for: Molecular insights into ROS-induced aggregation in chicken primordial germ cells and its alleviation by ascorbic acid
Source: J Anim Sci Biotechnol. 2026 Nov 9;17:204. doi: 10.1186/s40104-026-01521-9 (PMC13746570; doi:10.1186/s40104-026-01521-9)
Supplement: Supplementary file 2 [file 40104_2026_1521_MOESM1_ESM.docx]

**Fig. S1** Number of DEGs and proteins DEPs identified across the four breeds. **A** DEG counts across different groups. **B** DEP counts across different groups

**Fig. S2** Dose-response assessment of three antioxidant interventions on PGC viability. **A-C** Dose-response assays of SFN, AA, and GSH-EE in WC PGCs. Cell viability, and total cell counts were measured following a 48 h treatment with varying concentrations of each antioxidant. All data are presented as the mean ± SD (*n* = 3 independent biological replicates). Statistical significance was evaluated using Dunnett's test compared with the untreated control group (^*^*P* < 0.05, ^**^*P* < 0.01, and ^***^*P* < 0.001)
